# Crocetin Downregulates the Proinflammatory Cytokines in Methylcholanthrene-Induced Rodent Tumor Model and Inhibits COX-2 Expression in Cervical Cancer Cells

**DOI:** 10.1155/2015/829513

**Published:** 2015-03-22

**Authors:** Bing Chen, Zhao-Hui Hou, Zhe Dong, Chun-Dong Li

**Affiliations:** Department of Obstetrics and Gynaecology, Air Force General Hospital, PLA, Beijing 100142, China

## Abstract

The effect of crocetin (C_20_H_24_O_4_) on methylcholanthrene- (MCA-) induced uterine cervical cancer in mice was studied in this paper. After the mice were treated orally with crocetin, maleic dialdehyde (MDA), polymorphonuclear cells (PMN), interleukin-1*β* (IL-1*β*), and tumor necrosis factor-*α* (TNF-*α*) were examined by ELISA or immunohistochemistry. The inducible nitric oxide synthase (iNOS) activation in *HeLa* cells was analyzed using fluorescence microscopy for light microscopic examination. The MCA mice showed a significant increase in plasma MDA, PMN, IL-1*β*, TNF-*α*, and nitrates levels. At the same time, the mRNA level of COX-2 in *HeLa* cells was also significantly increased. These changes were attenuated by crocetin supplementation in the MCA mice. Crocetin supplementation in the MCA mice also showed protection against cervical cancer. These results suggest that crocetin may act as a chemopreventive and an anti-inflammatory agent.

## 1. Introduction

Cervical cancer is the second most common cancer in women worldwide [1]. Despite improved knowledge of the etiology of cervical cancer, aggressive cytoreductive surgery, and modern combination chemotherapy, there has been little change in the mortality statistics over the last 30 years. Compelling evidence has shown that the majority of cancers arise from sites of chronic irritation, infection, and inflammation, solidifying the concept that chronic unabated inflammation is critical for tumour progression [2]. The microenvironment of the tumor highly resembles an inflammation site which results from enhancement of the levels of cytokines, chemokines, neutrophils, eosinophils, mast cells, lymphocytes, and macrophages both in the surrounding stroma and within the neoplasm itself [3]. Recent clinical trials have shown that long-term anti-inflammatory treatment can be beneficial in colorectal cancer [4]. So the pharmacological agents effective for the treatment of inflammatory diseases may also be employed in cervical cancer.

Crocetin (C_20_H_24_O_4_) is one of the major active constituents of saffron, which is derived from the dried stigma of* Crocus sativus* L., and belongs to the Iridaceae family. Previous studies have demonstrated various pharmacological effects of this active constituent including its antioxidant, anti-inflammatory, and antitumor effects on some cell lines and animal models of cancer [4–8]. However, nothing is known about effects of crocetin in the uterine cervix tumorigenesis. In the present study, we investigated its anti-inflammatory effect on the interleukin-1*β* (IL-1*β*), tumor necrosis factor-*α* (TNF-*α*), and PMNs activity in a methylcholanthrene- (MCA-) induced uterine cervix tumorigenesis murine model system. Further, the effects of crocetin on expression of cyclooxygenase-2 (COX-2) in HeLa cells were also evaluated.

## 2. Material and Methods

### 2.1. Drugs

Methylcholanthrene (MCA) was purchased from Sigma Co., Ltd. Crocetin was purchased from Yiji Natural Products Co., Ltd., China.

### 2.2. Animals

This study was performed in accordance with the Guide for the Care and Use of Laboratory Animals. Care was taken to minimize discomfort, distress, and pain of the animals.

### 2.3. Experimental Design

Female Kunming strain mice weighing 20–22 g were maintained at room temperature under alternating natural light/dark photoperiod and had access to standard laboratory food and fresh water* ad libitum.* Murphy's string method [9] was followed for the induction of tumors in the uterine cervix of mice. Briefly, sterile double cotton thread impregnated with beeswax containing 600 *μ*g of MCA was inserted into the canal of the uterine cervix by means of laparotomy under mild ether anaesthesia. Forty-eight of these mice were allocated equally into 4 groups: MCA-induced group, MCA and Crocetin-10 group, MCA and Crocetin-20 group, and MCA and Crocetin-40 group. The other 12 normal mice were used as the control group. From then on, the 5 groups of mice were administered orally saline, Crocetin 10 mg/kg, Crocetin 20 mg/kg, Crocetin 40 mg/kg, and saline, respectively. Crocetin was dissolved in distilled water and administrated orally twice daily using a feeding needle for 35 days, and control group received double distilled water instead of crocetin.

At the end of the experimental period, the animals were fasted overnight (18 h) and then sacrificed by decapitation, the blood was collected to be centrifuged at 3000 rpm for 20 min, and the clear serum was separated for the measurement of inflammatory cells and inflammatory mediators. The tumor volumes and iNOS protein histochemistry were determined.

### 2.4. Measurement of Maleic Dialdehyde (MDA)

MDA was determined with thiobarbituric acid (TBA) using the manufacturer's instructions (Nanjing Jiancheng Bioengineering Institute). Total protein content of the samples was analyzed using Coomassie blue assay (Nanjing Jiancheng Bioengineering Institute).

### 2.5. Measurement of Infiltration of PMN

Myeloperoxidase (MPO) activity was measured to assess the extent of PMN infiltration. The method of assaying MPO activity was according to the guide of the assay kit (Nanjing Jiancheng Bioengineering Co. Ltd., China).

### 2.6. Measurement of IL-1*β* and TNF-*α* Level

The concentration of IL-1*β* and TNF-*α* was determined using a commercial ELISA kit (Shanghai Jinma Biological Technology, Inc., China) following the manufacturer's instruction.

### 2.7. Assessment of iNOS Protein Histochemistry

The tumors were dissected free from soft tissues at the end of the study and collected on chrome gel subbed slides. Tissue sections were fixed in 50% acetone in phosphate-buffered saline for 3 minutes at room temperature and endogenous peroxidase activity was blocked by 1.5% H_2_O_2_ in methanol for 15 minutes. The iNOS protein was determined using a commercial ELISA kit (Shanghai Jinma Biological Technology, Inc., China) following the manufacturer's instruction.

### 2.8. Cell Culture

HeLa cells (human cervical cancer HeLa cell line was obtained from Cell Culture Center of Shanghai Science Academy, China) were maintained in Petri dishes at 37°C in a Dulbecco-modified Eagle culture medium supplemented with 10% fetal calf serum and 1% antibiotic mixture, under a humidified atmosphere containing 5% carbon dioxide. The culture medium was changed twice per week. After they had reached their growth plateau, the cells were used for inoculation by mechanical harvest and transfer to fresh culture medium.

### 2.9. Crocetin Inhibited COX-2 Production in HeLa Cells

Analysis of COX-2 production was performed using an ELISA kit according to manufacturer's instructions (Shanghai Jinma Biological Technology, Inc., China). HeLa cells were treated with various concentrations of crocetin (1, 10, 50, and 100 *μ*mol/L) for 24 h. Cell culture supernatants were collected to measure the concentration of COX-2. Production of COX-2 was normalized to protein concentrations.

Total RNA was extracted from crocetin-treated and untreated HeLa cells using GenElute Mammalian Genomic Total RNA Isolation Kit (Sigma) as per the manufacturer's protocol. The extracted RNA samples were then subjected to reverse transcription- (RT-) PCR for analysis of expression of COX-2.

### 2.10. Statistical Analysis

The data were expressed as mean ± SEM and results were analyzed by ANOVA followed by Dunnett's* t* test. *P* < 0.05 was considered significant.

## 3. Results and Discussion

A considerable body of evidence has supported the concept that tumors can originate at the sites of infection or chronic inflammation [10–12]. Many pathological disorders or diseases, including cervical cancer, are characterised by the exacerbated activation and maintenance of inflammatory pathways [13, 14]. In the current study, we found that MCA-induced carcinogenesis in the mouse uterine cervix in animals results in significantly increased circulating concentrations of inflammatory cytokines and tumor necrosis factor-*α* (TNF-*α*), when compared with controls. It is in agreement with previous data [10–14].

**Figure 1:**
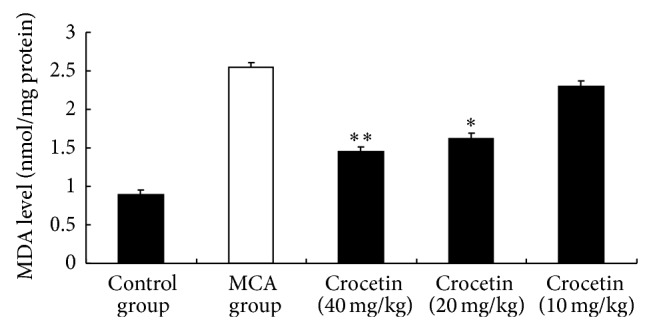
Effect of crocetin on MDA level. Values represent the mean ± SEM. ^*^ *P* < 0.05 versus MCA group. ^**^ *P* < 0.01 versus MCA group.

Peroxidation damage plays an important role in the progression of inflammation mediated diseases in particular cervical cancer. The central dogma in chronic inflammation hypothesis emphasizes the role of ROS generated by phagocytes, which cause cytotoxicity and mutagenesis [15–17]. Therefore, the antioxidant effects of crocetin were investigated by measuring MDA levels. The control animals showed low MDA levels; however, the MDA levels in the MCA group were significantly higher (*P* < 0.05). As shown in Figure 1, MDA levels in the crocetin (40 mg/kg) and crocetin (20 mg/kg) groups were significantly lower than those in the MCA group (*P* < 0.01 and *P* < 0.05, resp.). This result is in agreement with previous reports [18, 19]. It suggests that crocetin might exert a profound effect on inhibition of lipid peroxidation and free radical generation.

The association between tumor cells and polymorphonuclear cells (PMNs) has been demonstrated in several types of cancer [20, 21]. However, the role of PMNs in cervical cancer progression has not been well studied in vivo. In this study, the PMNs activity was relatively low in control group and significantly increased in the MCA group. Treatment with crocetin of 40 mg/kg significantly reduced PMNs activity (Table 1). Treatment with crocetin of 20 mg/kg reduced PMNs activity also. However, it is not significant. PMNs may contribute to secondary injury by causing microvessel occlusion and releasing oxygen radicals, cytolytic proteases, and proinflammatory cytokines, which may induce the neuronal damage [22, 23]. In the present study, we observed a dose-dependent inhibitory effect of crocetin on PMNs activity, indicating less neutrophil infiltration into the lesion site.

**Figure 2:**
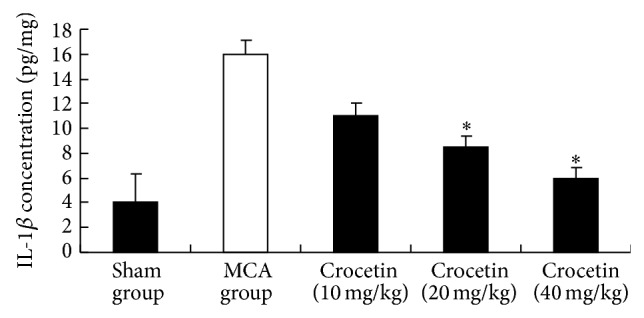
Effect of crocetin on IL-1*β* concentration. Values represent the mean ± SEM. ^*^ *P* < 0.05 versus MCA group.

**Figure 3:**
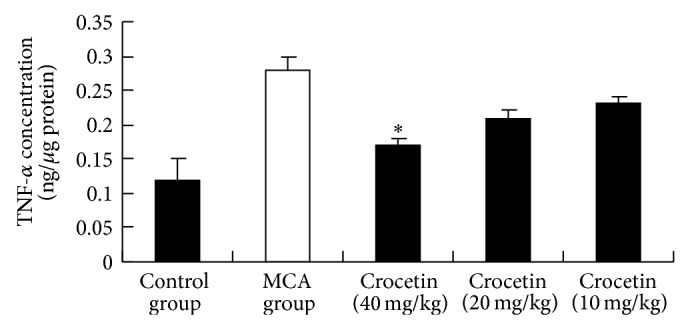
Effect of crocetin on TNF-*α* concentration. Values represent the mean ± SEM. ^*^ *P* < 0.05 versus MCA group.

Key features of cancer-related inflammation include the infiltration of white blood cells and cytokines such as interleukin (IL)-1, IL-6, and tumor necrosis factor-*α* (TNF-*α*) [24]. IL-1*β* has been shown to be upregulated in many cancers and confers chemoresistance in pancreatic carcinoma [25]. Our study is consistent with these studies.  Figure 2 shows that MCA significantly increased protein concentration of IL-1*β*. Crocetin (40 mg/kg) and crocetin (20 mg/kg) treatment decreased the level of IL-1*β* by 34% and 56% as compared to the MCA group, respectively (*P* < 0.05). TNF-*α* is an inflammatory cytokine which may play important roles in the progression of cervical lesions [26]. TNF-*α* was measured in blood to evaluate whether the concentration of this cytokine was systemically influenced by crocetin. Furthermore, this helps elucidate whether or not TNF-*α* can be implicated in the effect that was seen on neutrophil migration. As shown in Figure 3, the levels of TNF-*α* elevated significantly after induction of tumors with MCA. Crocetin (40 mg/kg) suppressed this response (*P* < 0.05). We demonstrated in this study that administration of crocetin decreased serum levels of IL-1*β* and TNF-*α* that are known to be produced by induction of tumors with MCA. Results from studies on cytokines have given us some insight into the mechanisms involved in the protection of crocetin against cervical cancer (Table 2).

**Figure 4:**
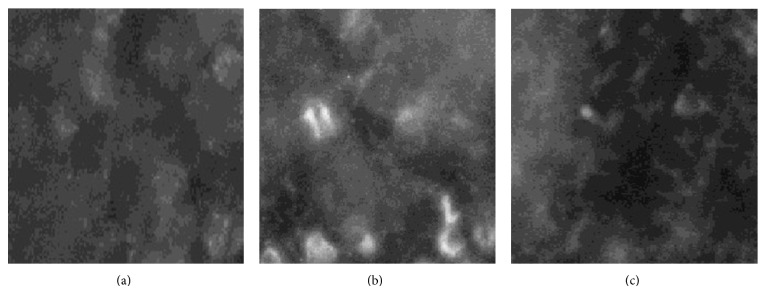
Demonstration of iNOS protein in tumor. All photographs were taken at an exposure time of 1 s. Magnification ×400. (a) iNOS in cytoplasm of tumor cells of control rat. (b) iNOS in cytoplasm of tumor cells of IMO rat. (c) iNOS in cytoplasm of tumor cells of crocetin (40 mg/kg) rat.

Inducible NOS is induced in response to inflammatory-like stimuli and is capable of sustained production of high levels of NO that predominate during inflammation [27]. The excessive or inappropriate production of NO can damage tissue through the superoxide anion (O_2_^−^) [28]. The fluorescence microscopy analyses showed evidence of widespread iNOS expression in tumor cells from animals with MCA but no evidence of iNOS immunoreactivity in controls. Treatment with crocetin (40 mg/kg) also inhibited the iNOS expression in bone marrow cells (Figure 4).

**Figure 5:**
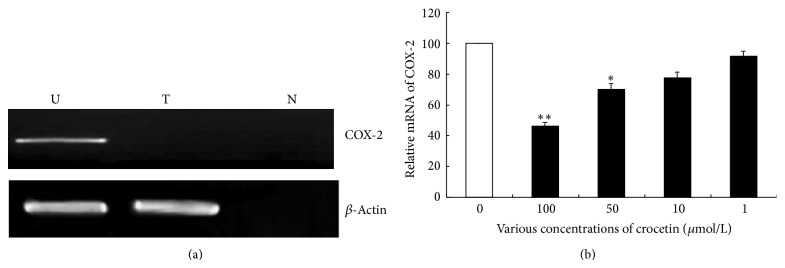
Crocetin inhibited COX-2 production and expression in HeLa cells. (a) Expression analysis of COX-2 in HeLa cells. Lane U shows untreated HeLa cells; lane T shows crocetin-treated HeLa cells; lane N shows negative control for RT-PCR. *β*-Actin was used as an internal control. Representative gels from one of the three experiments were used. (b) The mRNA level of COX-2 was measured in HeLa cells treated with various concentrations of crocetin for 24 h. Values represent the mean ± SEM. ^*^ *P* < 0.05 versus control group.

According to a previous research by Subbaramaiah and Dannenberg, COX-2 is involved in inflammation and its upregulation has been reported in various cancers [29]. In the present study, HeLa cells were shown to express high levels of COX-2, which was subsequently downregulated on treatment with crocetin (Figure 5(a)). Relative to nontreated controls, crocetin dose-dependently decreased COX-2 production in cervical cancer cells (Figure 5(b)). It has been suggested that COX-2 is an important target for the chemopreventive effects of these agents. The present study is consistent with this study [30]. Our observations prompt us to suggest that inhibition of COX-2 production by crocetin may suppress growth and invasiveness of cervical carcinomas.

## 4. Conclusion

This study suggested for the first time that crocetin has an anti-inflammatory effect by suppressing the level of IL-1*β* and TNF-*α* as well as PMNs activity in a MCA-induced uterine cervix tumorigenesis murine model system. Further, crocetin dose-dependently decreased COX-2 production in cervical cancer cells. Overall, crocetin demonstrated potent in vitro and in vivo anti-inflammatory activities. It provided support for the potential of using crocetin as a chemopreventive and an anti-inflammatory agent.

## Figures and Tables

**Table 1:** Effects of crocetin on PMNs activities.

Different groups	(*μ*mol·g^−1^)
Control	0.99 ± 0.20
MCA	2.00 ± 0.20
Crocetin (40 mg/kg)	1.00 ± 0.10^*^
Crocetin (20 mg/kg)	1.30 ± 0.25
Crocetin (10 mg/kg)	1.80 ± 0.22

Values are shown as means ± SEM. ^*^ *P* < 0.05 versus MCA group.

**Table 2:** Effects of crocetin on tumor volumes.

Different groups	Tumor volume (mm^3^)
Control	0
MCA	10.4 ± 0.30
Crocetin (40 mg/kg)	6.00 ± 0.10^*^
Crocetin (20 mg/kg)	7.32 ± 0.22
Crocetin (10 mg/kg)	8.80 ± 0.33

Values are shown as means ± SEM. ^*^ *P* < 0.05 versus MCA group.
